# *Solanum dulcamara* L. Berries: A Convenient Model System to Study Redox Processes in Relation to Fruit Ripening

**DOI:** 10.3390/antiox12020346

**Published:** 2023-02-01

**Authors:** Milica Milutinović, Đura Nakarada, Jelena Božunović, Miloš Todorović, Uroš Gašić, Suzana Živković, Marijana Skorić, Đurđa Ivković, Jelena Savić, Nina Devrnja, Neda Aničić, Tijana Banjanac, Miloš Mojović, Danijela Mišić

**Affiliations:** 1 Institute for Biological Research “Siniša Stanković”—National Institute of the Republic of Serbia, University of Belgrade, 11060 Belgrade, Serbia; 2 Faculty of Physical Chemistry, University of Belgrade, 11158 Belgrade, Serbia; 3 Innovation Centre of Faculty of Chemistry Ltd., University of Belgrade, 11158 Belgrade, Serbia

**Keywords:** *Solanum dulcamara*, bittersweet, fruits, redox state, ROS, antioxidants, EPR imaging

## Abstract

The present study provides, for the first time, a physicochemical and biochemical characterization of the redox processes associated with the ripening of *Solanum dulcamara* L. (bittersweet) berries. Electron Paramagnetic Resonance Spectroscopy (EPRS) and Imaging (EPRI) measurements of reactive oxygen species (ROS) were performed in parallel with the tissue-specific metabolic profiling of major antioxidants and assessment of antioxidant enzymes activity. Fruit transition from the mature green (MG) to ripe red (RR) stage involved changes in the qualitative and quantitative content of antioxidants and the associated cellular oxidation and peroxidation processes. The skin of bittersweet berries, which was the major source of antioxidants, exhibited the highest antioxidant potential against DPPH radicals and nitroxyl spin probe 3CP. The efficient enzymatic antioxidant system played a critical protective role against the deleterious effects of progressive oxidative stress during ripening. Here, we present the EPRI methodology to assess the redox status of fruits and to discriminate between the redox states of different tissues. Interestingly, the intracellular reoxidation of cell-permeable nitroxide probe 3CP was observed for the first time in fruits or any other plant tissue, and its intensity is herein proposed as a reliable indicator of oxidative stress during ripening. The described noninvasive EPRI technique has the potential to have broader application in the study of redox processes associated with the development, senescence, and postharvest storage of fruits, as well as other circumstances in which oxidative stress is implicated.

## 1. Introduction

Despite the natural profusion of antioxidants in fruits that can process reactive oxygen species (ROS) and benefit human health, it is surprising that our fundamental knowledge about redox processes in fruits is still fragmentary. Progress in understanding the molecular mechanisms involved in redox regulation and the role of ROS during fruit ripening and senescence could help us to develop new strategies to optimize fruit nutritional quality, production, and storage [1]. On the other hand, a detailed characterization of redox processes in fruits is important to understand the molecular and biochemical basis of fruit ripening, since, during this process, the ratio between oxidized and reduced species can be disturbed by various endogenous (e.g., hormonal) and environmental (abiotic and biotic stress) factors.

ROS are molecules produced during oxygen metabolism; some of the most widely analyzed ROS in plants are singlet oxygen, superoxide, hydrogen peroxide, and hydroxyl radicals. The three main sources of ROS in plants are chloroplastic photosynthesis, mitochondrial respiration, and the peroxisomal photorespiration cycle. Photosynthesis functionally accommodates redox reactions in plants that are underpinned by the transfer of electrons between a donor and an acceptor, which, consequently, generates ROS. In tissues with low or no photosynthesis, such as fruits, ROS are preferentially produced in mitochondria, peroxisomes, and apoplasts [2]. It is known that the fruit ripening process is associated with increased oxidative stress, resulting from stimulated ROS production, which might result in the breakdown of cell wall components, membrane disruption, and cellular decompartmentation [3]. Cellular damage occurs when reduction–oxidation (redox) homeostasis becomes unbalanced, as a result of a disturbed equilibrium between the production and the scavenging of ROS, which is maintained by the antioxidant system, which includes enzymatic and nonenzymatic (antioxidants) components. Antioxidants include metabolites with antioxidant properties, which, in fruits, are profuse and variable in their diversity and quantity, and are affected by endogenous (developmental and genetic) and environmental (abiotic and biotic) factors. Enzymes of the antioxidant machinery rapidly process ROS, i.e., catalase (CAT; EC 1.11.1.21), superoxide dismutase (SOD; EC 1.15.1.1), guaiacol peroxidase (GX; EC 1.11.1.7), and ascorbate–glutathione cycle enzymes: ascorbate peroxidase (APX; EC 1.11.1.11), monodehydroascorbate reductase (MDHAR; EC 1.6.5.4), dehydroascorbate reductase (DHAR; EC 1.8.5.1), glutathione S-transferase (GST; EC 2.5.1.18), glutathione peroxidase (GPX; EC 1.11.1.9), and glutathione reductase (GR; EC 1.8.1.7). In addition, enzymes such as peroxidase or dismutase can be considered as both ROS-generating and ROS-processing components.

The present work aims to fill knowledge gaps on redox processes in fruits by tracking metabolic changes associated with fruit ripening in *Solanum dulcamara* L. (bittersweet), comparatively analyzing the tissue-specific profiles of antioxidants and activities of antioxidant enzymes in mature green (MG) and ripe red (RR) berries, in parallel with the assessment of the redox status of the fruits. The imaging of the redox status of fruits using Electron Paramagnetic Resonance (EPR) spectroscopy (EPRS) and imaging (EPRI) improved our understanding of redox state alterations during ripening, and the resulting consequences. Due to its fruit morphology, and relatedness with many crop species in the Solanaceae family (e.g., potato, tomato, eggplant, and pepper), *S. dulcamara* is a suitable model to use to study the cellular metabolism and regulatory cascades of oxidative stress/antioxidants guiding normal fruit ripening. Bittersweet has bright red berries in loose clusters of 3–20 that ripen in summer and fall. As the berries ripen on the hanging clusters, they turn from green to orange and are ripe when they are bright red. All stages of flower and berry development and ripening can be found on the same plant [2]. The thin-skinned berries are round to egg-shaped, 10–15 mm in length and 7.5–10 mm in width, and contain about 30 flattened yellow seeds. For the first time, the morphology of bittersweet berries allowed measurements of redox status adopting the EPR methodology on intact fruits. EPRI, as a non-invasive technique capable of detecting the levels of single molecules related to cellular metabolism and distinguishing biochemical/physiological alterations in a spatial and temporal manner, provided the means to characterize different fruit tissues with high specificity and sensitivity. EPRS and EPRI are experimental techniques known to provide valuable physiological information related to the evaluation of the ex vivo/in vivo levels and spatiotemporal distribution of antioxidative constituents (primary, secondary metabolites, and ROS) in animals [3] and in plants [4]. EPRS is considered the gold standard for such use since, unlike other spectroscopy methods, it does not depend on the optical properties of the system, has higher sensitivity, and has lower detection limits compared to other commonly used techniques. For this purpose, the EPR spin probe method with nitroxyl spin probes is often used, allowing the detection of its characteristic EPR signal over a prolonged period.

## 2. Materials and Methods

### 2.1. Metabolomics of MG and RR Bittersweet Fruits

#### 2.1.1. Chemicals and Reagents

Standards (HPLC-grade ≥ 98%) of 5-*O*-caffeoylquinic acid (chlorogenic acid, CGA), caffeic acid (CA), *p*-coumaric acid (*p*-CoA), quercetin-3-*O*-rutinoside (rutin, R), vitexin, quinic acid, aconitic acid, and abscisic acid were purchased from Sigma-Aldrich (Steinheim, Germany). Acetonitrile and formic acid (MS grade) were obtained from Merck (Darmstadt, Germany). Ultrapure water (MicroPure water purification system, 0.055 μS/cm) was used to prepare standard solutions and dilutions. Syringe filters (25 mm, PTFE membrane 0.45 μm) were purchased from Supelco (Bellefonte, PA, USA).

#### 2.1.2. Sampling and Storage of Bittersweet Berries

Bittersweet fruits of wild-growing *Solanum dulcamara* L. (accepted name, The World Flora Online (WFO)) plants were harvested in 2021 in Mirijevo locality, Belgrade (44°48′09.0′′ N 20°31′36.6′′ E), and selected based on the ripening stage and lack of pests, diseases, and mechanical damage. Maturity stage was determined based on a subjective indicator of covering color and uniformity in size (10 mm in length and 7.5 mm in width). Fruits of both maturity stages (mature green, MG; and red ripe, RR) were collected from one individual plant and stored at −80 °C or 4 °C, for further metabolic profiling and EPRI analysis, respectively. All phytochemical analyses were performed in triplicates, each representing the biological pool of three fruits. In parallel, fruits were subjected to manual dissection under the binocular microscope where the skin, pulp, and seeds were separated, flash-frozen in liquid nitrogen, ground into a fine powder using a mortar and pestle, and stored at −80 °C for further metabolic and enzyme activity measurements. At least 20 fruits per maturity stage (MG and RR) were harvested and pooled for each biological replicate. This was repeated three times, providing material from at least 60 different fruits.

#### 2.1.3. Preparation of Methanol Extracts of Bittersweet Berries

Samples (whole fruits and separated tissues) were diluted in 96% methanol (*v:w* = 10:1) and vortexed for 1 min. Extraction was performed overnight at 4 °C. The next day, extraction was continued in an ultrasonic bath (RK100, Bandelin, Berlin, Germany) at room temperature for 20 min and the samples were subsequently centrifuged at 8000× *g* for 20 min. Following filtration using 15 mm RC filters with a 0.22 µm pore size (Agilent Technologies, Palo Alto, CA, USA), supernatants were stored at 4 °C until use. All extractions were performed in biological triplicates.

#### 2.1.4. UHPLC-LTQ OrbiTrap MS Qualitative Analysis of Phenolics in Bittersweet Fruits

LC/MS analysis was performed using an Accela UHPLC system (Thermo Fisher Scientific, Bremen, Germany) coupled to an LTQ OrbiTrap mass spectrometer equipped with a heated electrospray ionization (HESI) probe (Thermo Fisher Scientific, Bremen, Germany). A Syncronis C18 column (100 × 2.1 mm, 1.7 µm particle size) from Thermo Fisher Scientific was used as the analytical column for separation. The chromatographic and MS settings were the same as previously described in the literature [5]. Tentative identification of unknown compounds was conducted based on their monoisotopic mass and MS^3^ fragmentation, and confirmed using previously reported MS fragmentation or data [6,7,8,9,10,11]. ChemDraw software (version 12.0, CambridgeSoft, Cambridge, MA, USA) was used to calculate the accurate mass of compounds of interest. Xcalibur software (version 2.1, Thermo Fisher Scientific, Waltham, MA, USA) was used for instrument control, data acquisition, and data analysis.

#### 2.1.5. UHPLC/DAD/(±)HESI−MS^2^ Quantification of Major Phenolics

Methanol extracts of the intact MG and RR bittersweet fruits, as well as of the separated skin, pulp, and seeds, were analyzed for the content of major phenolic compounds. Analyses were performed using the Dionex Ultimate 3000 UHPLC system (Thermo Fisher Scientific, Bremen, Germany) with a DAD detector, configured with a triple quadrupole mass spectrometer (TSQ Quantum Access MAX, Thermo Fisher Scientific, Basel, Switzerland). Samples were chromatographically separated on a Synchronis aQ C18 column (100 × 2.1 mm) with 1.7 µm particle size (Thermo Fisher Scientific, USA), thermostated at 40 °C. The mobile phase, consisting of water + 0.1% formic acid (A) and acetonitrile + 0.1% formic acid (B), was eluted according to the gradient previously described in [12]. The flow rate of the mobile phase was set to 0.3 mL min^−1^ and the injection volume was 10 μL. DAD absorption was acquired at λ_max_ = 260 and 320 nm. A triple quadrupole mass spectrometer with heated electrospray ionization (HESI) was operated in a negative ionization mode, with the following parameter settings: vaporizer temperature: 300 °C; spray voltage: 4000 V; sheet gas (N_2_) pressure: 26 AU; ion sweep gas (N_2_) pressure: 1.0 AU; auxiliary gas (N_2_) pressure: 10 AU; capillary temperature: 275 °C; skimmer offset: 0 V. Argon was used as the collision gas in the collision-induced fragmentation of the molecules, and collision energy (cE) was set to 30 eV. Each targeted compound was quantified by tracking two diagnostic MS^2^ fragments and an external standard method was employed for the quantification.

The calibration curves of all targeted compounds showed excellent linearity with correlation coefficients of *r*^2^ = 0.999, *p* < 0.001. Total concentrations of the analyzed phenolics were obtained by calculating the peak areas on MS chromatograms corresponding to methanol extracts of MG and RR fruits. They are expressed as μg per g of plant fresh/dry weight (μg g^−1^ FW). Xcalibur software (version 2.2) was used for the instrument control, data acquisition, and analysis.

### 2.2. EPR Measurements

All EPR spectra were recorded using a Bruker ELEXSYS-II X/L spectrometer (Rheinstetten, Germany) with either R4123SHQE X-band or ER540R23 L-band resonators. EPR spectra were analyzed using Xepr software (Bruker BioSpin). Hydrogen peroxide, methanol (MS grade), DPPH (2,2-Diphenyl-1-picrylhydrazyl), 3CP (3-Carbamoyl-2,2,5,5-tetramethyl-3-pyrrolin-1-oxyl), and 3CxP were purchased from Sigma-Aldrich (Steinheim, Germany). Spin trap DEPMPO (5-(Diethoxyphosphoryl)-5-methyl-1-pyrroline-N-oxide) was purchased from Focus Biomolecules (Plymouth Meeting, USA). AccuGENE deionized 18 MΩ water was from Lonza (Bornem, Belgium). Iron(II) sulfate heptahydrate (97%) was purchased from Merck (Darmstadt, Germany).

#### 2.2.1. Determination of the Scavenging Activity of Whole and Selected Tissue Bittersweet Extracts towards DPPH Radicals

The distinctive shape of the DPPH EPR spectra enables the determination of the initial radical concentration and the ability of bittersweet fruit methanolic (MeOH) extracts to reduce their presence in the system [13]. The DPPH reduction activity of extracts made from bittersweet fruits was studied using the previously described EPR methodology [13,14,15]. In brief, 1 µL of bittersweet fruit extracts (in methanol or water) were added to the 29 µL of the 210 µM DPPH solution in the appropriate solvent (methanol or water). The mixture was transferred into the gas-permeable Teflon tube, and the X-band EPR signal was recorded for 2 min upon the addition of the DPPH solution into the mixture, using the following experimental settings: microwave power: 10 mW; microwave frequency: 9.85 GHz; modulation frequency: 100 kHz; modulation amplitude: 2 G. Another set of experiments, performed under the same experimental settings and using the same DPPH concentration, was performed to study the reduction capacity of MeOH extracts made from excised bittersweet fruit tissues (skin, pulp, and seeds). Control recordings were made by substituting the samples with the same volume of the solvent. The antiradical activity of the extracts (AA) is expressed as:

$$AA=\frac{I_{c}-I_{a}}{I_{C}}100\;\left(\%\right)$$

where I_c_ and I_a_ refer to the double integral values of the control and samples determined from the EPR spectra, respectively. The data presented in this study are the mean values of three independent measurements. The unpaired Student’s t-test statistics were used for statistical comparisons. Differences at the level below *p* ≤ 0.05 were considered statistically significant.

#### 2.2.2. Determination of the Scavenging Activity of Bittersweet Fruits Water Extracts towards Hydroxyl Radicals

To evaluate the capacity of bittersweet fruits to remove ^•^OH radicals, a Fenton reaction containing the spin trap DEPMPO was deployed [16,17]. This spin trap was selected for its well-known good selectivity and long DEPMPO/OH spin adduct half-life (132 min) [18]. Since the spin adduct’s natural degradation process and influence on the potential interaction of the extract components with spin adducts could affect the EPR signal, it was important to obtain EPR spectra immediately after initiating the Fenton reaction. In brief, 30 µL of the sample which contained 23 µL of H_2_O, 3 µL of the whole bittersweet H_2_O extract, 2 µL of H_2_O_2_ (final concentration 0.35 mM), and 1 µL of DEPMPO (final concentration 3.5 mM) was transferred into the gas-permeable Teflon tube, and 1 µL of FeSO_4_ (final concentration 0.15 mM) was applied 2 min before the EPR spectra were acquired. Recordings were made using the following experimental settings: microwave power: 10 mW; microwave frequency: 9.85 GHz; modulation frequency: 100 kHz; modulation amplitude: 1 G. The antiradical activity of extracts (AA) is expressed the same way as in the previous experiments. The data presented in this study are the mean values of three measurements. The unpaired Student’s t-test statistics were used for statistical comparisons. Differences at the level below *p* ≤ 0.05 were considered statistically significant.

#### 2.2.3. Determination of the Capacity of Bittersweet Fruit Tissues to Reduce Pyrrolidine Spin Probes (X-Band 1D Gradient Imaging)

**Figure 1:**
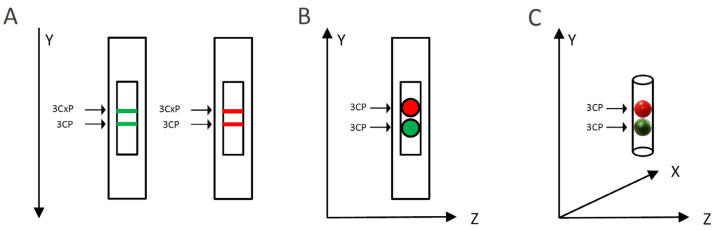
Schematic representation of the EPRI experimental setup for: (**A**) X-band 1D gradient, (**B**) X-band 2D, and (**C**) L-band 2D imaging. Green and red colors represent mature green (MG) and ripe red (RR) bittersweet fruits, respectively.

To evaluate the capacity of MG or RR bittersweet fruit samples to perform intra/extracellular reduction of pyrrolidine spin probes, membrane-permeable (3CP) and membrane-impermeable (3CxP) spin probes were used. For this purpose, 1 mm thick strips of MG and RR bittersweet fruits of the same mass (3.2 mg) were placed onto a quartz tissue cell, one above the other, and recorded simultaneously using an X-band 1D gradient EPR experiment (Figure 1A). One of the tissue samples was soaked with 5 μL of 0.1 mM 3CP and the other with 5 μL of 0.1 mM 3CxP, and these were recorded using a magnetic field gradient along the Y-axis, with the following parameters: microwave power: 10 mW; microwave frequency: 9.8 GHz; modulation frequency: 100 kHz; modulation amplitude: 2 G; magnetic field gradient: 22 Gcm^−1^. The value of the applied gradient was set to obtain the best nitroxides EPR peak signal separation from each tissue sample. The double integral values of the EPR signal reduction kinetics of each signal were measured for at least 70 min.

#### 2.2.4. Spatiotemporal Visualization of the Capacity of the Bittersweet Fruits to Reduce Pyrrolidine Spin Probe (X-Band 2D Imaging)

To visualize the capacity of MG and RR bittersweet fruits to perform the extra/intracellular reduction of the pyrrolidine spin probe, membrane-permeable (3CP) spin probes were used. For this purpose, 1 mm thick round slice samples of the same mass (15 mg), excised from MG and RR fruits, were placed onto a quartz tissue cell, one above the other, and recorded using X-band 2D imaging EPR experiment (Figure 1B). An RR fruit slice was soaked with 20 μL of 15 mM 3CP, an MG bittersweet tissue sample was soaked with 20 μL of 2 mM 3CP, and EPR signals were recorded using an imaging experiment in the ZY-plane with the following parameters: microwave power: 10 mW; microwave frequency: 9.8 GHz; modulation frequency: 100 kHz; modulation amplitude: 2 G; magnetic field gradient: 20 G cm^−1^. The 2D images were recorded at intervals of 10, 20, 30, 55, 95, and 115 min. To obtain a high-quality 2D EPR image during the prolonged-time period, a higher concentration of 3CP had to be used for the RR fruit samples since they showed a considerably higher spin probe reduction capacity.

#### 2.2.5. Spatiotemporal Visualization of the Capacity of the Intact Bittersweet Fruits to Reduce Pyrrolidine Spin Probe (L-Band 2D Imaging)

To visualize the capacity of the intact MG and RR bittersweet fruits to perform the extra/intracellular reduction of the pyrrolidine spin probe, membrane-permeable (3CP) spin probes were used. For this purpose, intact MG and RR fruits of approximately the same mass (325 mg) were placed one above the other and images were recorded using an L-band 2D imaging EPR experiment (Figure 1C). Before imaging, fruits were imbibed under vacuum with 100 mM of 3CP, and recorded using an imaging experiment in the ZY-plane, with the following parameters: microwave power: 10 mW; microwave frequency: 9.8 GHz; modulation frequency: 30 kHz; modulation amplitude: 2 G; magnetic field gradient: 20 G cm^−1^. The 2D images were made at intervals of 70, 90, 110, 140, 160, and 170 min.

### 2.3. High Performance Thin Layer Chromatography (HPTLC) and HPTLC-DPPH Bioautography Assay

On the glass HPTLC plate silica gel 60 F_254_ (20 cm × 10 cm, Art. 105642, Merck), 3 µL of fruit sample extracts, or the methanol solution of standards, were applied as an 8 mm band (Linomat 5 CAMAG, Muttenz, Switzerland). HPTLC development was performed with ethyl acetate-toluene-formic acid-water (16:4:3:0.5, *v*:*v*:*v*:*v*) as a mobile phase, up to a migration distance of 80 mm in a 10 min saturated twin trough chamber followed by drying under a cold flow of air (hair dryer). An image of the HPTLC chromatogram was captured with a mobile phone—Huawei P20 lite—under 366 nm (DigiStore 2). For the derivatization of phenolic compounds, the HPTLC chromatogram was immediately immersed in derivatization solution (0.5% 2-aminoethyl diphenylborinate in methanol) and a 5% solution of PEG 400 in methanol, for 2 s with an immersion speed of 3.5 cm s^−1^, using Chromatogram Immersion Device III (CAMAG, Muttenz Switzerland). The image captured at 366 nm was saved as a TIF file. All the extracts of *S. dulcamara* fruits—as well as solutions of standards in 96% methanol, namely, CGA (1 mg mL^−1^), CA (0.5 mg mL^−1^), *p*-CoA acid (1 mg mL^−1^), and R (1 mg mL^−1^)—were applied on HPTLC plates at 3 µL.

For the HPTLC-DPPH assay, to detect radical scavengers as a yellow band against a violet background, the HPTLC chromatogram was subjected to post-chromatographic derivatization by dipping it into a 0.1% (*w*:*v*) methanol solution of DPPH, which was followed by drying at 60 °C for 1 min. The HPTLC chromatogram derivatized with DPPH solution was stored in the dark for 30 min, and images were subsequently recorded under white light. For the HPTLC-DPPH assay, 5 µL of each of the *S. dulcamara* fruit samples was applied on HPTLC plates. The solutions of CGA, CA, and R, were applied on HPLTC plates at 2 µL, while 3 µL of *p*-CoA (1 mg mL^−1^) was used.

### 2.4. Activities of Antioxidant Enzymes

Protein extraction was performed as described in [19]. Protein concentration was determined following the instructions in the Qubit protein assay kit (Fisher Scientific) using a Qubit 3.0 Fluorimeter (Life Technologies Corporation, NY, USA). The results were obtained using three biological replicates for two developmental fruit stages and three different tissues of fruit (skin, pulp, and seeds), while each biological replicate represented a pool of at least twenty fruit samples. Total CAT, POX, and SOD activity were determined as previously reported in [20]. Total APX activity was assayed spectrophotometrically (Agilent 8453 spectrophotometer, Life Sciences, NA, USA) following the method of [21]. The activities of antioxidant enzymes were presented in specific activity units per milligram of total protein content (U mg^−1^). PPO activity was measured using the method described in [22], with minor modifications. The initial rate of the absorbance change was determined at 410 nm for 4 min at 20 s intervals using an ELISA microplate reader (Multiskan FC, Thermo Scientific, Waltham, MA, USA).

The content of hydrogen peroxide (H_2_O_2_) was determined using the method described in [23], with slight modifications. Briefly, powdered fruit samples (0.2 g) were directly homogenized with 1 mL of a solution containing 0.25 mL of trichloroacetic acid (TCA), 0.5 mL of 1 M potassium iodide (KI), and 0.25 mL of potassium phosphate buffer (10 mM, pH 7), vortexed and subsequently centrifuged at 10.000× *g* for 20 min at 4 °C. The absorbance of the samples (supernatant) was measured at 390 nm (Agilent 8453 spectrophotometer, Life Sciences, NA, USA). The H_2_O_2_ content was determined using a standard curve of H_2_O_2_ and was expressed as μmolg^−1^ FW.

### 2.5. Statistical Analysis

All experiments were repeated three times, and the values presented here are the means of the three independent measurements. The results are expressed as means with standard error (± SE). Statistical significance was analyzed using Statgraphics Statistical Software (Statgraphics Technologies Inc., The Plains, VA, USA). For the statistical analysis of antioxidant enzyme activities, one-way ANOVA was performed, followed by Fisher’s LSD test. For the UHPLC/(−)HESI-MS^2^ quantitative analysis of major phenolic acids in MG and RR fruits, Student’s t-test was utilized, while for the UHPLC/(−)HESI-MS^2^ quantitative analysis of major phenolic acids in fruit sections (seed, pulp and skin), one-way ANOVA was performed followed by Fisher’s LSD pairwise test.

## 3. Results

### 3.1. Metabolic Profiling of Solanum Dulcamara Fruits

**Figure 2:**
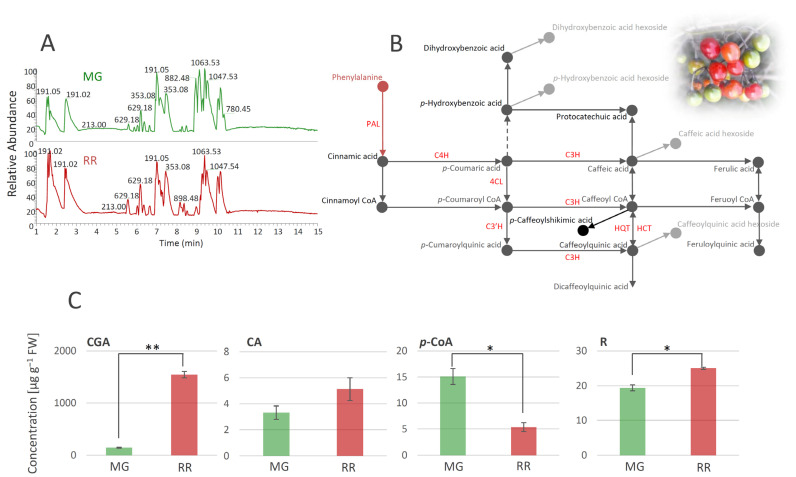
UHPLC/LTQ Orbitrap MS^n^ chromatograms of methanol extracts of mature green (MG) and ripe red (RR) bittersweet fruits (**A**). Based on the qualitative profiles of polyphenolics, a putative biosynthetic route of major phenolic acids in bittersweet fruits is proposed (**B**), which is mainly represented by chlorogenic acid (caffeoylquinic acid), caffeic acid, and *p*-coumaric acid. UHPLC/(−)HESI-MS^2^ quantitative analysis of major phenolic acids in MG and RR fruits (**C**): Asterisks (*) denote significant differences between MG and RR fruits according to the *t*-test, *p* values: * *p* ≤ 0.05; ** *p* ≤ 0.01. Abbreviations: PAL—phenylalanine ammonia-lyase; C4H—cinnamate hydroxylase; 4CL—4-hydroxycinnamoyl-CoA ligase; C3H—*p*-coumaroyl ester 3-hydroxilase; C3′H—*p*-coumaroylester 3′-hydroxylases; HQT—hydroxycinnamoyl CoA quinate transferase; HCT—hydroxycinnamoyl-CoA shikimate/quinate hydroxycinnamoil transferase; CGA—chlorogenic acid (caffeoylquinic acid); CA—caffeic acid; *p*-CoA—*p*-coumaric acid; R—rutin.

Using the untargeted LC/MS approach, a total of 83 metabolites were identified in extracts of *S. dulcamara* MG and RR fruits (Table 1, Figure 2A). Metabolites were divided into several groups according to their structural characteristics (Table 1). Thus, the below table lists **22** polyphenolic compounds (**19** derivatives of phenolic acids and **3** flavonoids), **10** amides, **16** saponins, **14** steroidal alkaloids, **6** lignans, and **15** other compounds.

Phenolic acids were mainly represented as hexosyl derivatives and esters with quinic acid. In addition, two free hydroxycinnamic acids, CA (compound **11**) and *p*-CoA (**12**), were identified. The presence of 5-*O*-caffeoylshikimic acid (**15**), which gives specific fragments of caffeic acid residues (179, 135, and 170 *m/z*), was confirmed by the literature data [27]. Some compounds from the group of amides, such as various derivatives of putrescine, spermidine, octopamine, and tyramine, are very common in the Solanaceae family [29,59,60]. All of the identified amides in *S. dulcamara* (Table 1), except pantothenic acid (**21**), belong to the subgroup of phenolic amides. It has previously been shown that pantothenic acid can be found in different parts of *Solanum phureja* [28]. As for flavonoids, all three detected compounds, quercetin-3-*O*-rutinoside (**30**), kaempferol-3-*O*-rutinoside (**31**), and dihydrocaempferol (**32**), have previously been reported in the Solanaceae family [8,32]. The presence of quercetin-3-*O*-rutinoside was confirmed by the available standard, while the other two compounds were tentatively identified by the comparison of their fragmentation data with the available literature (Table 1). A significant number of saponin derivatives were identified in our study. Saponins are a group of glycosides that contain triterpene or steroid compounds in addition to several sugar units, so their identification is quite difficult due to this complex structure. There is a large number of derivatives of the same mass with different arrangements of sugar units, and their exact structure cannot be solved using mass spectrometry alone. Thus, we named several compounds from this group as only ‘saponin derivatives’ (**33**–**35**, **37**, **43**, **47**, and **48**). Several saponin derivatives were named after the plant species from which they were first isolated. Thus, Soladulcoside A (**44**) was named after the plant that is the subject of this study [41]. Steroid alkaloids generally appear as corresponding glycosides in all plants of the genus *Solanum* and are very common in the Solanaceae family [9,10,46]. The presence of all **14** compounds from this subgroup of saponins was confirmed using the available literature. It should also be emphasized that a significant number of the listed derivatives were named after the genus *Solanum*, e.g., solanidenediol (**49**, **50**, **52**, and **53**), solanandaine (**51** and **55**), *β*-tomatine (**54**), *β*2-solasonine (**56**), *α*-solasonine (**57**), *γ*-solamarine (**58**), and *α*-solanine (**59**). Some of the alkaloids exhibit high toxicity toward humans or animal organisms. According to the literature, trisaccharides containing *α*- and *β*-solamarines were present in bittersweet berries, with higher amounts in the unripe tissues [10]. The amounts of steroidal alkaloids in bittersweet berries, identified in the present study, differed between MG and RR fruits (Table 1). MG fruits were a richer source of *γ*-solamarine, *α*-solasonine, *α*-solanine, abutiloside H, and solanandaine. On the other hand, solanidenediol triose derivatives, and leptines were much more abundant in RR fruits. From the group of lignans, two isomers of alangilignoside C were identified (**63** and **64**). Alangilignoside C was a common compound in the genus *Solanum*, and it was detected in the EtOH stem extract of *Solanum buddleifolium* [48]. Additionally, pinoresinol (**67**) and its hexosyl derivatives (**66**), isolariciresinol-3-*O*-hexoside (**65**), and syringaresinol (**68**) were found. The last group of compounds identified in *S. dulcamara* fruits was designated as ‘other compounds’, and includes a significant number of different derivatives that were already detected in *Solanum* species (compounds **72**, **73**, **76**, and **77**, which have a pentosyl-hexosyl moiety in their structure (Table 1)), with the exception of butanediolpentosyl-hexoside (**71**), which was previously found in the stems of *Acanthopanax senticosus* (Araliaceae). One camphor derivative, campherenane diol dihexoside malonate ester (**80**), which is characteristic of *Solanum habrochaites* [58], was identified in the MeOH extract. Compounds **81**–**83** were identified as octadecenoic acid derivatives [9]. Based on the polyphenolic profile of bittersweet berries, we were able to propose the metabolic network of the major compounds from this group (Figure 2B). The most abundant compounds belong to the group of phenolic acids, i.e., hydroxycinnamic acids, among which CGA and its derivatives predominate. *p*-CoA, CA, and their derivatives are also abundant. Among hydroxybenzoic acids, *p*-hydroxybenzoic acid prevailed, while R was the most abundant compound from the group of flavonoids. Further, we quantified the major phenolics (CGA, CA, *p*-CoA, and R), and traced the differences between MG and RR fruits (Figure 2C). It was observed that RR fruits represent a richer source of CGA, CA, and R than MG ones, while MG fruits possess more *p*-CoA than RR fruits.

### 3.2. Tissue-Specific Distribution of Polyphenolics in MG and RR Berries of S. dulcamara

**Figure 3:**
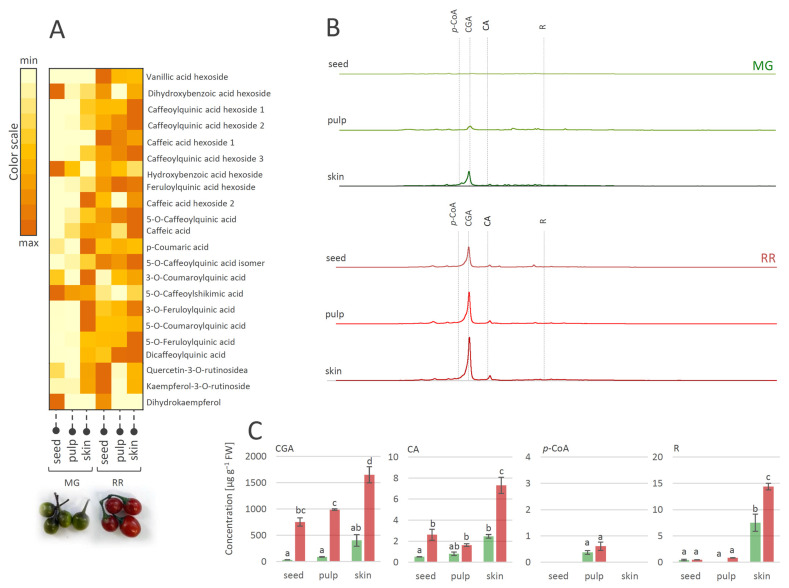
(**A**) Heat-map presenting relative amounts of polyphenolics in different tissues of mature green (MG) and ripe red (RR) bittersweet fruits, as analyzed by UHPLC/LTQ Orbitrap MS^n^. Color scale indicates values representing peak areas of compounds, distributed between min and max values among tissues, for each compound individually. (**B**) UHPLC/DAD chromatograms of MeOH extracts of seed, pulp, and skin of MG and RR fruits are presented, acquired at λ = 260 nm. (**C**) UHPLC/(−)HESI-MS^2^ quantitative analysis of major phenolic acids in MG and RR fruits. Letters above the bars denote significant differences according to Tukey’s pairwise test at *p* ≤ 0.05. Abbreviations: CGA—chlorogenic acid (caffeoylquinic acid); CA—caffeic acid; *p*-CoA—*p*-coumaric acid; R-rutin.

The fruit transition from the MG to the RR developmental stage obviously involves the qualitative and quantitative changes of metabolite profiles. These changes are tissue-specific and implicate modulations in the biosynthesis and accumulation of major antioxidant compounds from the group of phenolics. A number of studies on the phenolics contents of various *Solanum* species [61,62,63] reported that most of the phenolics were present in the fruit skin. Likewise, in this study, the MeOH-soluble metabolites of MG and RR fruits were analyzed and tissue-specific phenolic compounds in skin, pulp, and seeds were traced (Figure 3). Considering the qualitative content analysis of phenolics in MG and RR bittersweet fruits, polyphenolics were generally more abundant in the skin than in the pulp and seeds (Figure 3A). The exceptions were specific flavonoids (Table 1, compounds **30**, **31**, and **32**), and some phenolic acids (Table 1, compounds **2**, **7**, and **15**), which were more abundant in the seeds compared to the pulp and skin.

Bittersweet MG and RR fruits, especially their skin, were rich in hydroxycinnamic acids, mainly represented by CGA and its derivatives, CA, and flavonoid R (Figure 3B,C). On the other hand, targeted phenolics were much less abundant in fruit pulp, particularly in seeds, with the exception of *p*-CoA, which prevailed in the pulp of MG and RR fruits. When considering the differences between MG and RR fruits, it was observed that RR fruits contain more phenolics than MG. Thus, the amount of CGA in the skin of RR fruits reached approximately 1600 µg g^−^^1^ FW, while in the skin of MG fruits, ~400 µg g^−^^1^ FW was recorded. Other detected compounds were far less present in all analyzed fruit sections.

### 3.3. Tissue-Specific Redox State of Bittersweet Fruits

Fruit development comprises three main phases: cell division, cell expansion, and ripening. As green organs, young fruits possess photosynthetically active chloroplasts driving central metabolism, and hence, the ability to perform developmental processes [64]. During the ripening, chloroplasts become chromoplasts by losing green chlorophylls at the expense of colored antioxidants such as carotenoids [65,66]. Thus, fruit development is likely to present remarkable discrepancies in terms of redox signals, their source (e.g., chloroplastic, mitochondrial, peroxisomal, and apoplastic), and the duration and extent of oxidative stress [67].

#### 3.3.1. Scavenging Activity of MG and RR Bittersweet Fruits towards DPPH and Hydroxyl Radicals

**Figure 4:**
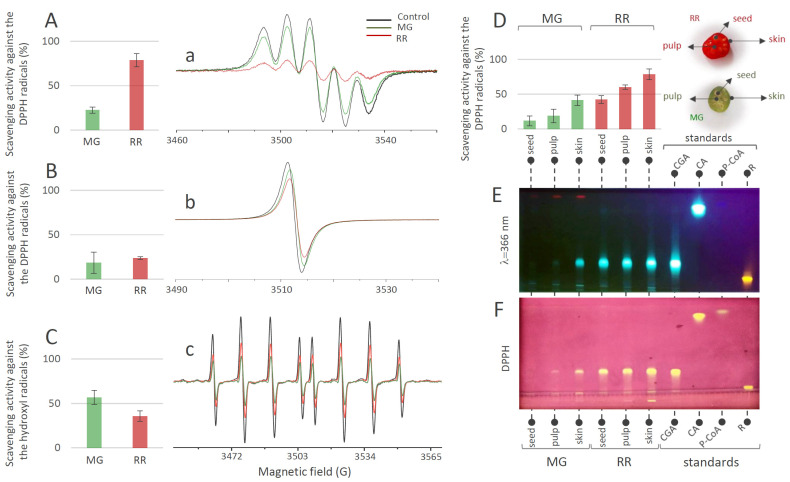
DPPH scavenging activity (%) obtained 2 min after the addition of DPPH into the bittersweet fruits system containing (**A**) MeOH extracts or (**B**) water extracts of MG (green bars) and RR (red bars), and representative EPR spectra of (**a**) DPPH radical in MeOH (black line), and in reaction with the MG (green line) and RR (red line) fruit MeOH extracts system, and (**b**) DPPH radical in H_2_O (black line), and in reaction with the MG (green line) and RR (red line) fruit water extracts system. (**C**) ^•^OH scavenging activity (%) obtained 2 min after the ^•^OH generation in the Fenton reaction system containing H_2_O extracts of MG (green) and RR (red) bittersweet fruits, and (**c**) representative EPR spectra of DEPMPO/OH spin adducts obtained in the Fenton reaction system without (black line) and in reaction with the MG (green line) and RR (red line) fruit water extracts. (**D**) DPPH scavenging activity (%) recorded 2 min after the addition of DPPH into the system containing MeOH extracts of the skin, pulp, and seeds of MG (green) and RR (red) bittersweet fruits. Values are the means of three biological replicates, and error bars represent signal-to-noise ratio. (**E**) HPTLC of MeOH extracts of seed, pulp, and skin of MG and RR bittersweet berries at 366 nm, and (**F**) corresponding HPTLCDPPH assay.

The results of the DPPH scavenging activity of MeOH extracts of fruits (Figure 4A) indicated that both MG and RR fruits possess significant potential for the elimination of this organic radical. This potential obviously increased with the progress in the ripening of the bittersweet fruit, since RR fruits displayed higher DPPH scavenging activity than MG. The results of the same experiment, when using H_2_O extracts of MG and RR bittersweet fruits (Figure 4B), indicated that most of the samples show similar DPPH scavenging potential, which goes slightly in favor of RR fruits. These results were significantly lower than the ones obtained for MeOH extracts, indicating that H_2_O extracts were less potent DPPH scavengers. This could be attributed to the difference in the polyphenolic profiles of the two types of extracts. Based on the obtained results, all samples showed a remarkable scavenging effect toward ^•^OH radicals (Figure 4C). It should be noted that, in contrast to DPPH scavenging experiments, H_2_O extracts of bittersweet fruits had to be diluted 10 times before applying the ^•^OH scavenging assay, since the scavenging effect was too intense. Interestingly, higher ^•^OH scavenging activity was recorded for the MG (56.76%) than for the RR fruits (35.75%).

#### 3.3.2. Tissue-Specific Scavenging Activity of MG and RR Bittersweet Fruits Extracts towards DPPH Radicals

The experiments involving MeOH extracts of the skin, pulp, and seeds of MG and RR fruits, and their DPPH scavenging activity (Figure 4D), revealed the existence of a gradient in DPPH scavenging potential: seed ˂ pulp ˂ skin. When considering the difference between MG and RR fruits, the RR fruit displayed significantly higher scavenging potential than the MG fruit. The antioxidant activities of the MG and RR fruits were positively correlated with the tissue-specific content of phenolic acids in MG and RR bittersweet fruits. Phenolic-rich RR fruits displayed a higher level of antioxidant activity than MG, and among the analyzed tissues, the phenolic-enriched fruit skin was the most potent antioxidant. It can be presumed that CGA is the major contributor to the overall antioxidant activity of MG and RR berries. This was supported by the HPTLC-DPPH autoradiography assay, which confirmed the existence of a gradient in CGA content and corresponding DPPH scavenging activity between the outer and inner tissues of bittersweet fruits, with the skin being the most potent antioxidant (Figure 4E,F). Rutin was proved to be the second most important antioxidant compound in bittersweet fruits, especially in the skin of both MG and RR fruits (Figure 4F).

#### 3.3.3. Determination of the Capacity of Bittersweet Fruits to Reduce Pyrrolidine Spin Probes (X-Band 1D Gradient Imaging)

**Figure 5:**
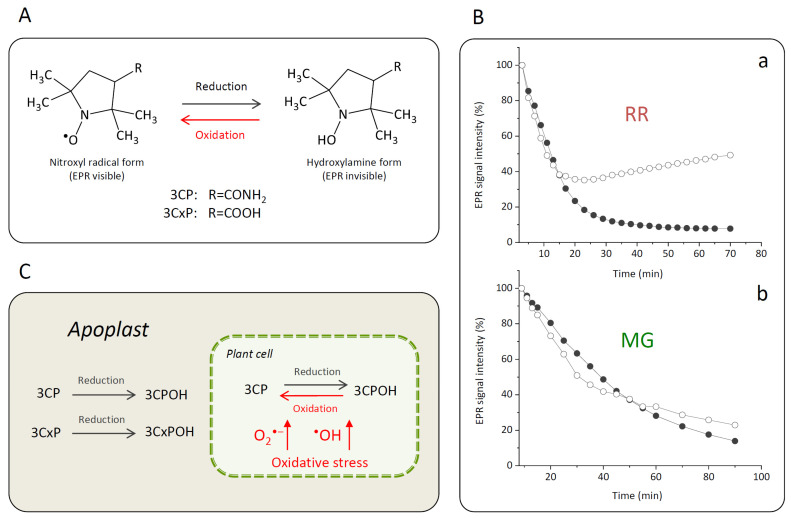
(**A**) Reversible one-electron reduction–oxidation showing the interconversion between the nitroxide 3CP and 3CxP spin probes (EPR visible) and corresponding reduced hydroxylamine forms 3CPOH and 3CxPOH (EPR invisible). (**B**) Kinetics of change of the EPR signal intensity of spin probes 3CP (white circles) and 3CxP (black circles) in strips of RR (**a**) and MG (**b**) bittersweet fruits adopting the X-band 1D gradient EPRI technique. (**C**) Proposed mechanism of the 3CP and 3CxP reduction–oxidation as influenced by the oxidative stress in bittersweet fruits and their localization in the cellular compartmentation.

When the nitroxyl spin probe was administered to the plant tissue, the ex vivo/in vivo EPR signal intensity of the probe was decreased by a one-electron reduction of the probe to the corresponding hydroxylamine (Figure 5A). The decay rate of the EPR signal intensity in a specific part of a plant was affected by several factors such as the concentration and distribution of probes and ROS, lipid solubility and membrane permeability of the spin probes, enzymatic/chemical one-electron reduction of nitroxyl radical, and reoxidation of the hydroxylamine back to the EPR active nitroxyl radical [3,68]. Accordingly, the proper selection of spin probes is an important issue for a successful EPR experiment. For instance, introduced in vivo and under strong redox conditions, piperidine nitroxides have a half-life of just a few minutes, whereas the half-life of pyrrolidine nitroxides is typically around 15 min or more. For the current investigation, two pyrrolidine spin probes, neutral membrane-permeable 3CP and negatively charged membrane-impermeable 3CxP, were used (Figure 5A). The hydrophilic spin probe 3CxP enables the measurement of radicals only outside the membranes, in the apoplasts of plant tissues, while the highly lipophilic spin probe 3CP also allows intracellular redox status measurements [69]. The combination of membrane-permeable spin probe 3CP and membrane-impermeable 3CxP provided us with kinetic information about physiologically relevant variations in ROS concentration, enabling high spatiotemporal resolution in various tissues (skin, pulp, and seed) of MG and RR bittersweet fruits. Based on the presented results obtained using thin strips of MG and RR bittersweet fruits (Figure 5Ba,Bb), both samples showed a significant reducing effect on spin probes 3CP and 3CxP. Likewise, compared to the MG bittersweet fruit, the RR fruit showed more intense 3CP and 3CxP radical reduction. This correlates with the results showing that the RR bittersweet fruits held an increased concentration of antioxidants compared to the MG ones, including CGA, CA, and R, which made RR fruits stronger radical scavengers. It is especially interesting that, in the case of RR fruits treated with spin probe 3CP (Figure 5Ba), the EPR signal intensity of this cell-permeable compound, after an initial decrease, started to increase after 20 min, which indicated the reoxidation of hydroxylamine in the corresponding nitroxyl spin probe. Since the applied concentrations of both spin probes—3CP and 3CxP—were the same, it could be concluded that the RR bittersweet fruits held a higher concentration of intracellular oxidative species than extracellular (apoplastic). A similar trend also existed in MG fruits (Figure 5Bb), but in a considerably less pronounced manner. One of the explanations for these rather complex spin clearance rate results, mainly observed after 20–30 min, could relate to the primary depletion of antioxidant species in observed samples, making visible a strong oxidative environment originating from H_2_O_2_-, O_2_^•−^-, and ^•^OH-induced oxidative stress through Fenton-like and/or metal-catalyzed Haber–Weiss reactions. In this study, the intracellular reoxidation of cell-permeable 3CP was recorded for the first time in some plant tissues. This phenomenon has previously been shown for animal/human model systems in which diamagnetic hydroxylamine can revert to corresponding paramagnetic nitroxyl radicals [3,70,71]. It has also been reported that hydroxylamines can be oxidized to nitroxides in the presence of oxidants (e.g., H_2_O_2_ and transition metal complexes), or if the cellular oxygen status permits [3,70,71]. According to the presented results, in bittersweet fruits, the bioreduction of cell-permeable nitroxides to the corresponding hydroxylamines and their reoxidation back to nitroxides is supported primarily by intracellular processes, and this is more pronounced in tissues of RR berries. It can be presumed that oxidative stress, resulting from a high intracellular O_2_^•−^ and/or ^•^OH environment, was more pronounced in RR bittersweet fruits (Figure 5C). Thus, the intensity of 3CP reoxidation can serve as a reliable indicator of intracellular oxidative stress in fruits during ripening. The present study shows that it is obvious that membrane-impermeable spin probes participated in the extracellular (apoplast) redox reactions of fruits, and were thus less sensitive to tissue redox status.

#### 3.3.4. Spatiotemporal Visualization of the Capacity of the Bittersweet Fruits to Reduce Pyrrolidine Spin Probe (X-Band 2D Imaging)

**Figure 6:**
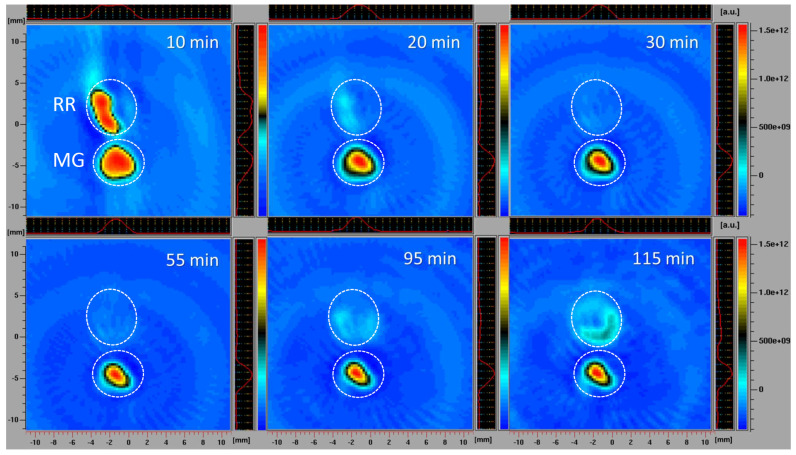
X−band 2D EPR images of slices of MG and RR bittersweet fruits recorded 10, 20, 30, 55, 95, and 115 min after the administration of the spin probe. The upper signal relates to the ripe red (RR) and the lower to the mature green (MG) bittersweet fruits. The signal amplitude in arbitrary units is shown in the form of color scales, which represent the intensities/concentrations of the 3CP spin probe in samples, with the red color indicating the zones/tissues with the highest content of 3CP in the form of nitroxyl radical. The black/green colors indicate tissues with lower levels of 3CP in the form of nitroxyl radical. MG fruits were treated with 2 mM 3CP, and RR fruits with 15 mM 3CP.

The simultaneous monitoring of spatiotemporal ROS dynamics in MG and RR bittersweet berries, using the EPR X-band mode, was a specific challenge. Different ripening stages of fruits may differ in several physiological properties, including concentrations of antioxidants and ROS, oxygen status, reducing capability, and pH. All these parameters could at least partially be responsible for the different redox states recorded in the MG and RR bittersweet fruits. The differences between intracellular and extracellular pH, for example, may have a profound influence on the uptake of exogenous molecules (spin probes). In the present study, slices of RR fruits were soaked with a 7.5 times higher concentration of 3CP compared to MG, which enabled the comparative spatiotemporal visualization of reduction capacity in bittersweet MG and RR fruits over a prolonged period. All samples showed a significant reducing effect on spin probe 3CP (Figure 6). The presented 2D images showed that the EPR signal of the 3CP from the RR fruits almost completely disappeared after 20 min, but then reappeared after 95 min, again indicating the reoxidation of this spin probe. This result additionally confirmed the presence of a higher amount of extra/intra-cellular antioxidants, and a higher concentration of oxidative species in RR than MG bittersweet fruits. The spatial distribution of the spin probe EPR signal illustrates that the inner tissues (seed and pulp) had lower free radical scavenging potential than the outer tissues (skin) of MG and RR bittersweet fruits.

#### 3.3.5. Spatiotemporal Visualization of the Capacity of the Intact Bittersweet Fruits to Reduce Pyrrolidine Spin Probe (L-Band 2D Imaging)

**Figure 7:**
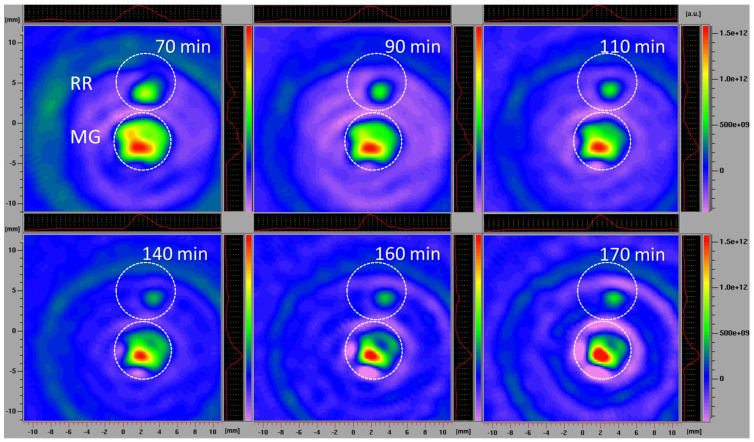
L−band 2D EPR images of MG and RR bittersweet fruits recorded after 70, 90, 110, 140, 160, and 170 min. The upper signal relates to the RR and the lower to the MG fruits. The signal amplitude in arbitrary units is shown on color scales, which present the intensities/concentrations of the 3CP spin probe in samples, with red color indicating the zones/tissues with the highest content of 3CP in the form of nitroxyl radical. The light/dark green colors indicate tissues with lower levels of 3CP in the form of nitroxyl radical.

The L-band 2D EPR imaging of intact fruits confirmed the imaging results obtained using fruit slices, which excluded the possibility that the process of preparing and handling tissue samples affected the results. The disadvantage of L-band EPR spectroscopy, compared to the X-band, is its lower sensitivity, requiring the use of higher spin probe concentrations. However, employing the L-band allowed for the study of intact fruit samples, instead of detached tissues, while still obtaining highly instructive images. A similar methodology was adopted to investigate the localization and distribution of paramagnetic species in the seeds of *Zanthoxylum limonella* [72], black soybean [73], and apple [74], but also in the roots of intact maize plants [4]. All these studies revealed that L-band 2D imaging is an excellent choice to visualize the distribution of free radicals in tissues with a satisfactory spectral resolution. Herein, in comparison to the previously performed X-band 2D experiments, a higher concentration of spin probe was adopted, and was equal in MG and RR fruits. Based on the presented 2D EPR images of MG and RR bittersweet fruits (Figure 7), all samples showed a significant reducing effect on the spin probe 3CP, demonstrating the presence of extra/intra-cellular antioxidants. From the very start of the experiment, RR fruit images displayed a low-intensity EPR signal, which gradually decreased until 140 min, and afterwards, its reappearance was observed up to 170 min. Thus, the intact RR fruit samples showed an initial high spin clearance rate, probably induced by a high concentration of antioxidants, and afterward, the effect of the high concentration of oxidative species prevailed. On the other hand, the spin probe EPR signal from the MG fruit was more intense and was constantly dropping from the start of the experiment until 160 min. A slight increase in the EPR signal was then observed up to 170 min, showing an initial lower spin clearance rate than in RR fruits, probably due to a lower concentration of antioxidants, and afterwards, a mild prooxidation effect. Again, the spatial distribution of the spin probe EPR signal illustrated that the inner tissues (seed and pulp) have lower free radical scavenging potential than the outer tissues (skin) of MG and RR bittersweet fruits. The literature data for animal/human model systems revealed that hypoxic conditions [75,76], tissue redox status [77,78], and oxidative stress accompanying the generation of hydroxyl radical [79,80] and/or superoxide radical [81,82] enhanced the conversion of paramagnetic nitroxide radicals to the corresponding diamagnetic products. Oxidizing conditions, such as exposure to hydrogen peroxide, inhibited the in vivo reduction of nitroxide radical [3]. It could be presumed that differences in the 3CP reduction rates between MG and RR bittersweet berries, were at least partially determined by the oxygenation status, i.e., the basal *p*O_2_ values of the two physiological states of fruits. More specifically, the observed decreasing antioxidants gradient (as a consequence of ROS concentration), from outer (skin) to inner fruit tissues (pulp and seeds), most probably followed the gradient in the oxygen concentration, which was the result of the low porosity of the fruits, limiting gas diffusion. Thus, our future work will aim to adopt EPR oximetry to describe tissue-specific oxygen concentration profiles in MG and RR fruits, in order to further uncover the changes in redox processes influenced by fruit ripening.

### 3.4. Tissue-Specific Activity of Antioxidant Enzymes in MG and RR Bittersweet Berries

**Figure 8:**
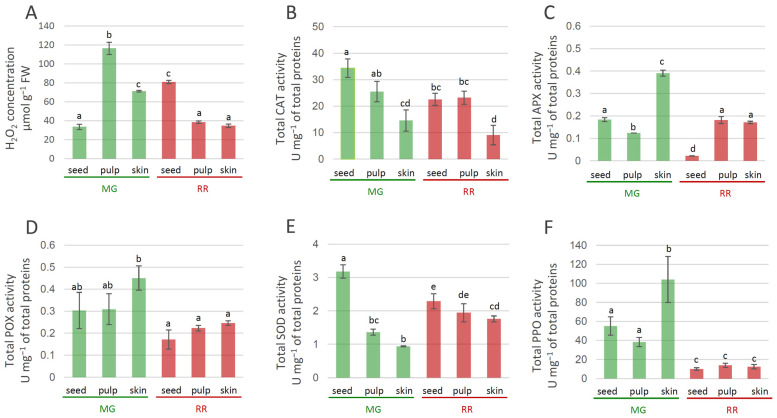
Tissue-specific concentration of H_2_O_2_ in MG (green bars) and RR (red bars) bittersweet fruits (**A**), as well as activities of antioxidant enzymes: CAT (**B**), APX (**C**), POX (**D**), SOD (**E**), PPO (**F**). Values are presented as means of three biological replicates and standard errors. Samples denoted by the same letter are not significantly different (*p* ≤ 0.05) according to Fisher’s LSD test. Abbreviations: CAT—catalase; APX—ascorbate peroxidase; POX—peroxidase; SOD—superoxide dismutase; PPO—polyphenoloxidase.

The fruit transition from MG to RR involved decreased levels of H_2_O_2_ in the skin and pulp at the later ripening stages of bittersweet fruits (Figure 8A). In seeds, differences in the levels of H_2_O_2_ between MG and RR fruits were also recorded, with increased H_2_O_2_ levels in RR seeds. Thus, H_2_O_2_ concentration displayed tissue-specific variations, with the pulp of MG fruits being the richest in H_2_O_2_ (Figure 8A). Previous studies have demonstrated that H_2_O_2_ levels increased in the Kyoho grape [83], mango [84], peach [85], and muskmelon [86] during fruit ripening. H_2_O_2_ is a strong oxidant that can initiate localized oxidative damage leading to the disruption of metabolic functions and loss of cellular integrity at sites where it accumulates [87]. To determine whether the increased oxidative stress accompanying bittersweet fruit ripening was associated with the reduced ability to enzymatically catabolize ROS, changes in the activities of SOD, CAT, POX, and APX, as well as of PPO, were determined to be a function of fruit ripening. Superoxide dismutases (SODs) and metalloenzymes are believed to play a crucial role in antioxidant defense [88] because they catalyze the dismutation of O_2_^•−^ to H_2_O_2_, while the removal of H_2_O_2_ is taken care of by CAT and/or POX and the enzymes of the ascorbate–glutathione pathways. The activity of CAT in MG and RR fruits was higher in inner tissues (seeds and pulp) than in skin (Figure 8B). The highest values of CAT activities were recorded in the seeds of MG bittersweet fruits. The activity of APX varied between tissues of MG and RR fruits, and was the highest in the skin of MG fruits (Figure 8C). In addition, the skin and pulp of RR fruits displayed higher levels of APX activity than seeds. The decrease in levels of APX activity found in the skin of bittersweet fruits during the ripening was also found for olive [89], tomato [90], and pepper fruits [91]. POX activity was generally higher in MG than in RR berries, with the skin of MG fruits displaying the highest values (Figure 8D). However, no significant variation in POX activities was recorded between different tissues of MG and RR fruits. A decreasing gradient in SOD activity from the inner (seeds) to outer parts (skin) of both MG and RR fruits was recorded (Figure 8E). SOD activity in the pulp and skin of RR fruits was higher than the activities in the corresponding tissues of MG fruits. The results indicate that MG fruits are more efficient than RR in detoxifying H_2_O_2_. Therewith, one can speculate that APX and POX are preferentially responsible for this process in the skin of MG bittersweet fruits, while CAT activity predominates in the seeds and pulp. Taking into account that SOD activity was the highest in the seeds of MG fruits, which also displayed the lowest H_2_O_2_ concentration, it could be presumed that enzymatic antioxidant machinery for H_2_O_2_ elimination was the most efficient in these organs. As for PPO, MG fruits generally displayed significantly higher activity than RR fruits (Figure 8F). The highest PPO activity was recorded in the skin of MG bittersweet fruits, which was followed by the seed and pulp of MG fruits. Similarly, unripe fruits of *Solanum lycocarpum* displayed higher levels of PPO activity than ripe [92]. It can be presumed that the lower content of phenolics in MG bittersweet fruits, when compared to RR, was at least partially a result of more active PPOs, which catalyzed the oxidation of phenolics to quinones. However, lower amounts of phenolics in MG fruits could also be a result of the lower intensity of their biosynthesis. Quinones react non-enzymatically with oxygen in the air to produce brown pigments in wounded tissues [93], which cause instant but differential browning in the fruits of a number of species, including crops belonging to the Solanaceae family [94,95]. It could be presumed that MG bittersweet fruits possessed higher potential for tissue browning than RR.

## 4. Conclusions

Bittersweet berries are a rich source of antioxidants, such as phenols, which scavenge ROS and thus increase the antioxidant capacity of the fruits. The major antioxidant in bittersweet fruits was CGA, which played a role in plant defense, and was susceptible to oxidation by PPO.

The MeOH and H_2_O extracts of bittersweet fruits possessed significant potential to eliminate DPPH radicals, which increased as ripening progressed. Both MG and RR bittersweet fruits showed a remarkable scavenging activity against ^•^OH radicals, with MG fruits being more efficient. The DPPH scavenging activity of the MeOH extracts was tissue-specific, displaying a decreasing gradient from the outer (skin) to the inner (pulp and seeds) tissues of the MG and RR bittersweet fruits, which may be related to the content of major antioxidants in the different tissues of the fruits, since the skin was the major site of phenolic accumulation.

The X-band 1D gradient EPR imaging results showed that all bittersweet tissue samples had a significant reducing effect on spin probes 3CP and 3CxP. Compared to the MG fruits, the RR fruits proved to be more active, which correlates with their higher concentration of antioxidants. Both the MG and RR fruits showed a high concentration of intracellular oxidative species, compared to extracellular oxidative species, in RR fruits in a more highly pronounced manner, and in MG fruits in a considerably less pronounced manner, probably due to H_2_O_2_-, O_2_^•−^-, and ^•^OH-induced oxidative stress by Fenton-like and/or metal-catalyzed Haber–Weiss reactions.

The X-band 2D EPR imaging of the bittersweet tissue samples confirmed the presence of a higher amount of extra/intra-cellular antioxidants, as well as a higher concentration of oxidative species in RR compared with MG fruits. The spatial distribution of the spin probe EPR signal illustrated that the seed and pulp had lower free radicals scavenging potential than the skin of thee MG and RR bittersweet fruits.

The L-band 2D EPR imaging of intact fruits was a convenient tool to monitor the conversion rate of suitable nitroxides to the corresponding diamagnetic species in order to evaluate the redox status of fruits in a non-invasive manner.

Herein, for the first time, the intracellular reoxidation of hydroxylamine to nitroxyl spin probe 3CP, confirmed by X-band 1D gradient EPRI, X-band 2D EPRI, and L-band 2D EPRI methodologies, was observed in some plant samples, and was highlighted as a reliable indicator of oxidative stress in fruits during ripening.

The results presented here suggest that, in the early stages of fruit ripening, an efficient antioxidant system comprising CAT, APX, POX, and SOD protected bittersweet fruits from the deleterious effects of progressive oxidative stress. The results indicated a shift to a severe cellular oxidative status during ripening and suggested a role for antioxidants in the process. It can be presumed that oxidative stress was more pronounced in RR fruits, partly due to the decreased activities of some of the ROS scavenging enzymes (POX, APX, and CAT).

## Figures and Tables

**Table 1:** High-resolution MS data and negative ion MS^3^ fragmentation of *Solanum dulcamara* metabolites.

No	Compound Name	*t*_R_, min	Molecular Formula, [M–H]^–^	Calculated Mass, [M–H]^–^	Exact Mass, [M–H]^–^	Δ mDa	MS^2^ Fragments, (% Base Peak)	MS^3^ Fragments, (% Base Peak)	Ref
**Phenolic acids**									
**1**	**Vanillic acid hexoside**	3.46	C_14_H_17_O_9_^–^	329.08781	329.08420	3.61	123(48), **161**(100), 169(22), 282(24), 283(30), 284(24), 285(37)	59(9), 71(15), 85(12), 97(11), **101**(100), 113(38), 143(19)	[24]
**2**	**Dihydroxybenzoic acid hexoside**	5.74	C_13_H_15_O_9_^–^	315.07216	315.06883	3.33	108(11), 109(12), 152(43), **153**(100), 154(8), 163(9), 165(12)	81(10), 108(9), **109**(100)	[9]
**3**	**Caffeoylquinic acid hexoside 1**	5.96	C_22_H_27_O_14_^–^	515.14063	515.13512	5.51	179(4), **191**(100), 192(7), 323(6), 341(7), 353(87), 354(11)	**85**(100), 93(47), 109(23), 111(40), 127(91), 171(20), 173(55)	[7]
**4**	**Caffeoylquinic acid hexoside 2**	6.47	C_22_H_27_O_14_^–^	515.14063	515.13454	6.09	179(8), 191(31), **323**(100), 324(15), 341(26), 353(21), 455(6)	133(5), **161**(100)	[7]
**5**	**Caffeic acid hexoside 1**	6.50	C_15_H_17_O_9_^–^	341.08781	341.08383	3.98	135(6), 161(27), **179**(100), 180(4), 203(5), 281(3)	107(20), **135**(100)	[6]
**6**	**Caffeoylquinic acid hexoside 3**	6.64	C_22_H_27_O_14_^–^	515.14063	515.13471	5.92	179(4), **191**(100), 192(6), 341(7), 353(49), 354(7), 395(14)	**85**(100), 93(43), 109(20), 111(33), 127(84), 171(28), 173(61)	[7]
**7**	**Hydroxybenzoic acid hexoside**	6.78	C_13_H_15_O_8_^–^	299.07724	299.07399	3.25	**137**(100)	**93**(100)	[9]
**8**	**Feruloylquinic acid hexoside *^b^***	6.87	C_23_H_29_O_14_^–^	529.15628	529.15155	4.73	**191**(100), 192(7), 193(8), 365(6), 367(79), 368(11), 409(12)	**85**(100), 93(52), 109(17), 111(32), 127(76), 171(18), 173(63)	[25]
**9**	**Caffeic acid hexoside 2**	6.87	C_15_H_17_O_9_^–^	341.08781	341.08375	4.06	135(8), **179**(100), 180(8)	107(22), **135**(100)	[6]
**10**	**5-*O*-Caffeoylquinic acid *^a^***	6.92	C_16_H_17_O_9_^–^	353.08781	353.08312	4.68	179(3), **191**(100), 192(3)	85(99), 93(64), 109(23), 111(41), **127**(100), 171(31), 173(68)	[7]
**11**	**Caffeic acid *^a^***	7.04	C_9_H_7_O_4_^–^	179.03498	179.03301	1.98	**135**(100)	91(68), **107**(100), 117(59), 135(72)	[26]
**12**	***p*-Coumaric acid *^a^***	7.08	C_9_H_7_O_3_^–^	163.04007	163.03862	1.45	103(24), 118(14), **119**(100), 120(14), 121(19), 135(29), 136(26)	**91**(100)	[27]
**13**	**5-*O*-Caffeoylquinic acid isomer**	7.45	C_16_H_17_O_9_^–^	353.08781	353.08321	4.59	179(3), **191**(100), 192(4)	**85**(100), 87(19), 93(55), 111(31), 127(88), 171(28), 173(61)	[7]
**14**	**3-*O*-Coumaroylquinic acid**	7.69	C_16_H_17_O_8_^–^	337.09289	337.08872	4.17	135(3), 163(5), 173(32), 179(13), **191**(100), 192(3)	85(89), 87(21), 93(59), 111(33), **127**(100), 171(28), 173(64)	[8]
**15**	**5-*O*-Caffeoylshikimic acid**	7.72	C_16_H_15_O_8_^–^	335.07724	335.07352	3.73	135(28), 161(3), **179**(100), 180(7), 191(3)	107(18), **135**(100)	[27]
**16**	**3-*O*-Feruloylquinic acid**	7.97	C_17_H_19_O_9_^–^	367.10346	367.09929	4.17	173(22), **191**(100), 192(7), 193(7), 203(6)	85(87), 93(65), 109(23), 111(37), **127**(100), 171(27), 173(62)	[9]
**17**	**5-*O*-Coumaroylquinic acid**	8.11	C_16_H_17_O_8_^–^	337.09289	337.08928	3.61	135(3), 163(5), 173(4), 179(10), **191**(100), 192(3)	**85**(100), 87(19), 93(64), 111(31), 127(90), 171(21), 173(75)	[8]
**18**	**5-*O*-Feruloylquinic acid**	8.27	C_17_H_19_O_9_^–^	367.10346	367.09963	3.82	**191**(100), 193(3)	85(97), 93(58), 109(22), 111(36), **127**(100), 171(23), 173(68)	[9]
**19**	**Dicaffeoylquinic acid**	9.04	C_25_H_23_O_12_^–^	515.11950	515.11530	4.20	173(7), 179(4), 203(11), 255(5), 299(9), **353**(100), 354(10)	93(20), 135(10), **173**(100), 179(62), 191(30)	[7]
**Phenolic amides**									
**20**	***N*-Caffeoylputrescine 1**	4.87	C_13_H_17_N_2_O_3_^–^	249.12447	249.12179	2.68	**135**(100), 136(8), 175(5), 176(5), 207(24), 208(3), 249(4)	91(26), 93(36), 106(5), **107**(100), 117(22), 135(3)	[9]
**21**	**Pantothenic acid**	5.73	C_9_H_16_NO_5_^–^	218.10340	218.10075	2.65	**88**(100), 89(5), 129(6), 143(8), 144(3), 146(12), 173(10)	**59**(100)	[28]
**22**	***N*-Caffeoylputrescine 2**	5.86	C_13_H_17_N_2_O_3_^–^	249.12447	249.12186	2.61	**135**(100), 136(5), 207(17), 249(3)	79(45), 91(5), 93(93), **107**(100), 117(11), 135(25)	[9]
**23**	***N^1^*,*N^14^*-*bis*-Dihydrocaffeoylspermine (kukoamine A)**	6.03	C_28_H_41_N_4_O_6_^–^	529.30316	529.29671	6.45	**365**(100), 366(21), 367(3), 408(53), 408(11), 419(3), 511(3)	115(3), 121(35), 122(3), 137(7), **243**(100), 244(12), 323(6)	[7]
**24**	***N^1^*-Caffeoy-*N^14^*-dihydrocaffeoylspermine**	6.10	C_28_H_39_N_4_O_6_^–^	527.28751	527.28172	5.79	**366**(100), 391(12), 407(51), 420(4)	109(3), 115(4), 121(33), 137(10), **243**(100), 323(5)	[7]
**25**	***N*-Feruloyltyramine 1**	8.19	C_18_H_18_NO_4_^–^	312.12413	312.12114	2.99	135(66), 148(19), 176(15), 177(12), **178**(100), 270(11), 297(68)	**135**(100), 136(19)	[11]
**26**	***N*-Caffeoyloctopamine**	8.65	C_17_H_16_NO_5_^–^	314.10340	314.10003	3.37	135(57), 150(16), 152(20), **161**(100), 162(9), 178(24), 192(19)	**133**(100)	[29]
**27**	**Acetyl tryptophan**	8.84	C_13_H_13_N_2_O_3_^–^	245.09317	245.09135	1.82	74(3), 116(5), 201(3), **203**(100), 204(10)	116(40), 129(10), 142(17), **159**(100), 186(7)	[30]
**28**	***N*-Feruloyltyramine 2**	10.27	C_18_H_18_NO_4_^–^	312.12413	312.12173	2.40	135(63), 148(15), 176(15), 177(15), **178**(100), 297(56), 298(11)	93(20), **135**(100), 136(13), 160(8)	[11]
**29**	**Grossamide**	11.89	C_36_H_35_N_2_O_8_^–^	623.23989	623.23378	6.11	297(29), 432(9), **460**(100), 461(28), 486(16), 591(26), 592(10)	282(9), 283(18), **297**(100), 298(5), 323(5), 445(16), 446(3)	[31]
**Flavonoids**									
**30**	**Quercetin-3-*O*-rutinoside *^a^***	7.99	C_27_H_29_O_16_^–^	609.14611	609.14096	5.15	255(4), 271(7), 300(12), **301**(100), 302(10), 343(5)	151(83), **179**(100), 255(58), 257(12), 271(95), 272(25), 273(19)	[8]
**31**	**Kaempferol-3-*O*-rutinoside**	8.47	C_27_H_29_O_15_^–^	593.15119	593.14561	5.58	229(3), 255(3), 257(4), 285(15), **285**(100), 286(14), 327(3)	197(18), 199(19), 213(24), 229(53), 241(31), **257**(100), 267(43)	[8]
**32**	**Dihydrokaempferol**	9.85	C_15_H_11_O_6_^–^	287.05611	287.05401	2.10	201(3), 243(9), **259**(100), 260(9), 269(4)	125(60), 151(17), 165(11), 172(16), 173(33), **215**(100), 241(19)	[32]
**Saponins**									
**33**	**Saponin derivative 1 *^b^***	8.52	C_66_H_99_O_30_^–^	1371.62267	1371.62202	0.64	1048(21), 1064(40), 1065(14), **1210**(100), 1211(37), 1226(52)	755(8), 884(7), 901(55), 918(7), 1046(6), **1048**(100), 1064(78)	[33]
**34**	**Saponin derivative 2 *^b^***	8.71	C_54_H_81_O_23_^–^	1097.51741	1097.51286	4.55	773(13), 934(15), **936**(100), 937(26), 1050(9), 1051(5), 1078(8)	594(5), 611(38), 755(7), **773**(100), 774(9)	[34]
**35**	**Saponin derivative 3 *^b^***	8.83	C_66_H_99_O_30_^–^	1371.62267	1371.61563	7.04	1064(28), 1080(13), 1210(47), 1211(21), **1226**(100), 1227(51)	738(3), 756(10), 900(3), 902(28), 917(30), **1063**(100), 1080(94)	[33]
**36**	**Solanigroside Y6**	8.97	C_57_H_93_O_27_^–^	1209.59097	1209.58649	4.48	902(45), **1048**(100), 1049(47), 1050(29), 1064(65), 1065(37)	738(3), 755(20), 884(10), 886(4), **902**(100), 903(6)	[35]
**37**	**Saponin derivative 4 *^b^***	9.21	C_45_H_69_O_20_^–^	929.43877	929.43736	1.41	750(4), 767(4), **883**(100), 884(44), 911(7), 912(4), 914(4)	574(20), 720(7), **721**(100), 737(6)	[36]
**38**	**Agamenoside A**	9.21	C_56_H_91_O_28_^–^	1211.57024	1211.56963	0.61	756(6), 918(22), 919(9), 1050(38), 1051(15), **1080**(100), 1081(21)	756(17), **917**(100), 919(4)	[37]
**39**	**Scopoloside I**	9.30	C_45_H_71_O_20_^–^	931.45442	931.45039	4.03	733(23), 752(62), 753(21), 770(48), 771(24), **913**(100), 915(38)	500(17), 575(8), 708(11), **733**(100), 751(21), 869(30), 895(28)	[38]
**40**	**Indioside D**	9.38	C_51_H_83_O_23_^–^	1063.53306	1063.52670	6.37	756(17), 900(10), **902**(100), 903(16), 916(8), 918(46), 919(9)	593(20), 740(7), **755**(100), 757(4)	[39]
**41**	**Protodioscin**	9.51	C_51_H_83_O_22_^–^	1047.53815	1047.53540	2.75	756(16), 757(14), 884(9), 885(8), **902**(100), 903(77), 904(46)	576(7), 593(4), 738(18), 739(3), 740(3), **756**(100), 757(12)	[35]
**42**	**Melongoside N**	9.57	C_45_H_75_O_19_^–^	919.49080	919.48480	6.00	596(4), 756(8), **758**(100), 758(18)	434(6), **595**(100), 596(7)	[7]
**43**	**Saponin derivative 5**	9.59	C_45_H_73_O_18_^–^	901.48024	901.47764	2.60	738(3), 740(4), 741(3), **756**(100), 757(37), 758(16)	413(25), 432(21), **575**(100), 576(10), 593(80), 595(15), 738(7)	[40]
**44**	**Soladulcoside A**	9.90	C_39_H_61_O_15_^–^	769.40160	769.40011	1.48	431(3), 710(12), 711(4), 734(9), **752**(100), 753(34), 754(8)	413(81), 431(26), 546(61), 575(27), 589(38), 707(61), **734**(100)	[41]
**45**	**Solaviaside A**	9.97	C_51_H_85_O_21_^–^	1033.55888	1033.55842	0.46	870(19), 885(14), 886(60), 887(24), **888**(100), 889(43), 890(15)	561(20), 724(9), 725(5), **742**(100), 742(7)	[42]
**46**	**Solasodoside A**	10.64	C_51_H_81_O_21_^–^	1029.52758	1029.52354	4.04	737(8), 866(22), 867(9), **884**(100), 885(27), 886(33), 887(21)	557(15), 558(3), 719(3), 722(15), 737(33), **738**(100), 866(4)	[43]
**47**	**Saponin derivative 6**	10.88	C_39_H_63_O_14_^–^	755.42233	755.41563	6.70	696(16), 697(6), 720(11), 721(4), **737**(100), 739(40), 740(8)	512(11), 561(12), 694(10), 708(9), **720**(100), 721(21), 1472(17)	[44]
**48**	**Saponin derivative 7**	10.88	C_45_H_69_O_18_^–^	897.44894	897.44657	2.37	605(14), 607(3), 734(14), 735(5), **751**(100), 752(21)	425(16), 443(5), 587(23), **606**(100)	[40]
**Glycoalkaloids**									
**49**	**Solanidenediol triose derivative 1**	7.26	C_45_H_72_NO_18_^–^	914.47549	914.46822	7.27	**753**(100), 754(57), 755(27), 756(5), 768(3), 769(3)	247(35), 307(6), 500(7), 540(72), 552(14), 606(10), **606**(100)	[45]
**50**	**Solanidenediol triose derivative 2**	7.60	C_45_H_72_NO_17_^–^	898.48057	898.47411	6.46	540(5), 734(3), 750(3), **753**(100), 754(43)	**247**(100), 442(7), 557(14), 606(41)	[46]
**51**	**Solanandaine**	7.90	C_45_H_72_NO_16_^–^	882.48566	882.48345	2.21	**737**(100)	247(53), 306(7), 428(63), 525(68), 554(11), **590**(100)	[7]
**52**	**Solanidenediol triose derivative 3**	8.03	C_45_H_72_NO_18_^–^	914.47549	914.46579	9.70	751(16), 752(56), **753**(100), 754(10), 768(6), 768(5), 867(12)	607(4), **722**(100), 734(13), 735(3)	[45]
**53**	**Solanidenediol triose derivative 4**	8.16	C_45_H_72_NO_17_^–^	898.48057	898.47398	6.59	576(3), 737(37), **738**(100), 739(14)	574(34), **576**(100), 591(3), 592(3)	[46]
**54**	**β-Tomatine**	8.25	C_45_H_74_NO_17_^–^	900.49622	900.48694	9.28	577(12), 578(15), 736(5), 737(41), 738(19), **739**(100), 740(89)	**576**(100), 578(32)	[10]
**55**	**Solanandaine isomer**	8.36	C_45_H_72_NO_16_^–^	882.48566	882.48044	5.22	**736**(100), 737(54), 738(38), 740(14)	**247**(100), 307(26), 572(30), 589(24), 590(18), 1422(31)	[7]
**56**	**β2-Solasonine**	8.36	C_39_H_62_NO_12_^–^	736.42775	736.42544	2.31	**574**(100)	410(16), 410(14), 412(15), 454(18), 468(49), **483**(100), 509(78)	[10]
**57**	**α-Solasonine**	8.96	C_45_H_72_NO_16_^–^	882.48566	882.47982	5.84	720(9), **721**(100), 722(51), 723(28), 724(7), 736(4)	307(4), 497(6), 508(27), 515(14), 520(6), 556(4), **574**(100)	[7]
**58**	**γ-Solamarine**	9.13	C_39_H_62_NO_11_^–^	720.43284	720.42971	3.12	246(4), 247(17), 307(6), 358(6), 508(13), **574**(100), 575(12)	**179**(100)	[10]
**59**	**α-Solanine**	9.13	C_45_H_72_NO_15_^–^	866.49020	866.48811	2.09	**721**(100), 722(87), 722(50), 723(10)	247(24), 265(6), 568(6), **574**(100), 1373(5), 1393(4)	[9]
**60**	**Abutiloside H**	9.15	C_46_H_74_NO_17_^–^	912.49622	912.48906	7.16	**867**(100), 868(8)	**720**(100)	[47]
**61**	**Leptine II**	11.60	C_47_H_74_NO_17_^–^	924.49622	924.48626	9.96	763(33), **764**(100), 764(5), 779(4), 780(3), 878(3)	246(12), 437(56), 551(43), 616(11), **617**(100), 746(26), 746(94)	[9]
**62**	**Leptine I**	11.77	C_47_H_74_NO_16_^–^	908.50131	908.49170	9.61	550(4), 762(58), **763**(100), 861(5), 863(8), 881(3), 892(4)	**247**(100), 599(85), 617(65)	[9]
**Lignans**									
**63**	**Alangilignoside C**	7.93	C_28_H_37_O_13_^–^	581.22396	581.21837	5.59	233(13), 373(13), **419**(100), 420(19), 533(25), 535(26), 566(12)	223(4), 373(7), 389(3), **404**(100), 405(4)	[48]
**64**	**Alangilignoside C isomer**	8.44	C_28_H_37_O_13_^–^	581.22396	581.21959	4.37	179(7), **401**(100), 402(17), 419(7), 533(43), 534(13), 535(6)	205(16), 220(49), 235(61), 356(37), 368(13), **371**(100), 386(91)	[48]
**65**	**Isolariciresinol 3-*O*-hexoside**	8.61	C_26_H_33_O_11_^–^	521.20284	521.19756	5.28	341(12), 359(9), 473(10), 474(21), **475**(100), 476(31), 477(8)	191(31), 253(56), 331(31), 343(96), 407(38), **415**(100), 433(99)	[49]
**66**	**Pinoresinol 3-*O*-hexoside**	8.74	C_26_H_31_O_11_^–^	519.18719	519.18198	5.21	**357**(100), 358(17)	136(33), **151**(100), 175(3), 311(14), 342(8)	[50]
**67**	**Pinoresinol**	8.75	C_20_H_21_O_6_^–^	357.13436	357.13017	4.19	136(33), **151**(100), 175(3), 311(14), 342(8)	**136**(100)	[51]
**68**	**Syringaresinol**	8.78	C_22_H_25_O_8_^–^	417.15549	417.15102	4.47	151(16), 166(30), 179(13), **181**(100), 353(10), 371(15), 402(44)	**166**(100)	[52]
**Other compounds**									
**69**	**Quinic acid *^a^***	1.61	C_7_H_11_O_6_^–^	191.05611	191.04617	9.94	**85**(100), 87(15), 93(41), 111(60), 127(61), 171(27), 173(46)	**57**(100)	[31]
**70**	**Aconitic acid *^a^***	1.67	C_6_H_5_O_6_^–^	173.00861	173.00753	1.08	**111**(100), 112(9), 129(5), 143(7), 155(17)	**67**(100)	[6]
**71**	**Butanediol pentosyl-hexoside *^b^***	5.25	C_15_H_27_O_11_^–^	383.15589	383.15155	4.34	161(11), 191(10), **251**(100), 336(6), 337(11), 338(11), 346(11)	85(4), 97(3), 101(18), 113(15), **161**(100)	[53]
**72**	**Phenethyl pentosyl-hexoside**	6.46	C_19_H_27_O_10_^–^	415.16097	415.15567	5.30	173(9), **191**(100), 192(11), 235(12), 253(14), 367(6), 397(6)	123(24), 133(41), 136(72), **149**(100), 173(96), 176(35)	[54]
**73**	**Benzyl pentosyl-hexoside**	7.25	C_18_H_25_O_10_^–^	401.14532	401.14150	3.82	175(11), **191**(100), 269(13), 353(13), 354(14), 355(24), 379(16)	113(3), 127(3),148(20), **176**(100)	[55]
**74**	**Tuberonic acid hexoside**	7.37	C_18_H_27_O_9_^–^	387.16606	387.16206	4.00	163(46), 191(13), 205(50), 207(74), **223**(100), 247(49)	**164**(100), 179(22), 208(36)	[9]
**75**	**Roseoside A**	7.58	C_19_H_29_O_8_^–^	385.18679	385.18185	4.94	113(5), 143(4), **153**(100), 161(26), 179(4), 205(76), 223(59)	95(7), 97(14), 109(7), 111(5), 137(18), **138**(100)	[9]
**76**	**Methylbutyl pentosyl-hexoside**	7.66	C_16_H_27_O_10_^–^	381.17662	381.17180	4.82	161(6), 217(4), **249**(100), 250(4), 336(3)	83(7), 85(11), 99(12), 101(69), 113(20), 159(23), **161**(100)	[6]
**77**	**Hexenyl pentosyl-hexoside**	7.92	C_17_H_29_O_10_^–^	393.17662	393.17246	4.16	131(25), **149**(100), 173(16), 191(73), 347(12), 353(57), 355(21)	88(3), 89(89), 113(14), **131**(100), 134(6)	[56]
**78**	**Deacetylasperuloside**	8.01	C_16_H_19_O_10_^–^	371.09837	371.09353	4.84	121(4), 191(18), 192(12), 193(22), 231(6), **249**(100), 250(4)	85(27), 95(15), 99(11), 111(10), **113**(100), 175(14), 231(82)	[9]
**79**	**Abscisic acid *^a^***	8.65	C_15_H_19_O_4_^–^	263.12888	263.12637	2.51	147(10), **153**(100), 216(16), 217(23), 218(32), 219(46), 246(15)	105(4), 111(8), 123(4), 124(14), 136(15), **138**(100)	[57]
**80**	**Campherenane diol dihexoside malonate ester**	10.97	C_24_H_39_O_10_^–^	487.25487	487.24932	5.55	161(10), 221(16), 440(15), **441**(100), 442(4), 443(11)	143(11), 147(8), 161(47), 221(10), 237(3), 321(4), **381**(100)	[58]
**81**	**Trihydroxyoctadecenoic acid**	11.72	C_18_H_33_O_5_^–^	329.23335	329.22954	3.81	**171**(100), 172(9), 201(6), 211(15), 229(22), 293(14), 311(19)	125(62), **127**(100), 153(99), 171(3)	[9]
**82**	**9,10,11-Trihydroxy-12, 15-octadecadienoic acid**	11.89	C_18_H_31_O_5_^–^	327.21770	327.21369	4.01	**171**(100), 172(7), 183(10), 201(36), 213(10), 291(12), 309(34)	97(5), 123(3), 125(55), **127**(100), 153(95)	[9]
**83**	**9,12,13-Trihydroxy-10-octadecenoic acid**	12.54	C_18_H_33_O_5_^–^	329.23335	329.22951	3.84	**171**(100), 172(8), 201(81), 202(8), 275(18), 293(19), 311(28)	123(3), 125(71), **127**(100), 153(78)	[9]

*^a^*—Confirmed using standards; *^b^*—compounds identified for the first time in the Solanaceae family; *t*_R_—retention time; Δ mDa—mean mass accuracy.

## Data Availability

All data underlying the results are available as part of the article and no additional source data are required.
